# P-2154. Cryptosporidium in pregnancy and the impact upon pregnancy outcomes

**DOI:** 10.1093/ofid/ofae631.2308

**Published:** 2025-01-29

**Authors:** Shirley Shapiro Ben David, Noam Orivieto, Yaakov Segal, Limor Adler

**Affiliations:** Maccabi Healthcare Services, Tel Aviv, Tel Aviv, Israel; Maccabi Healthcare Services, Tel Aviv, Tel Aviv, Israel; Maccabi Healthcare Services, Tel Aviv, Tel Aviv, Israel; Maccabi Healthcare Services, Tel Aviv, Tel Aviv, Israel

## Abstract

**Background:**

Cryptosporidiosis, an infectious disease caused by the protozoan parasite Cryptosporidium, poses a significant threat to individuals with compromised immune systems. Over the past four years, a notable rise in the incidence of cryptosporidiosis has been observed in Israel, including among pregnant women. Despite this increase, little is known about the impact of cryptosporidiosis on obstetrical outcomes. The objective of this study was to evaluate the influence of Cryptosporidium infection on pregnancy outcomes.

**Figure 1:**
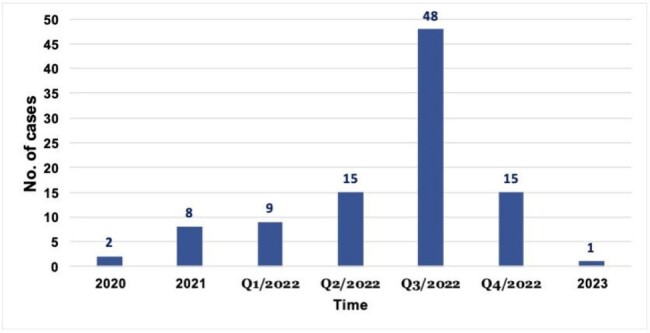
Epidemic curve, cryptosporidiosis in pregnant women, Maccabi Healthcare Services

**Methods:**

A retrospective case-control study. Pregnant women with positive results on multiplex real-time PCR stool tests for Cryptosporidium were compared to pregnant women with negative results, matched by age, week of pregnancy during the stool test, and sector. Medical records of the study cohort were reviewed for symptoms, treatments, pregnancy outcomes including termination, gestational age, birth weight, Apgar score, and head circumference. Significance was determined using Fisher's exact or Chi-square tests.

**Table 1:**
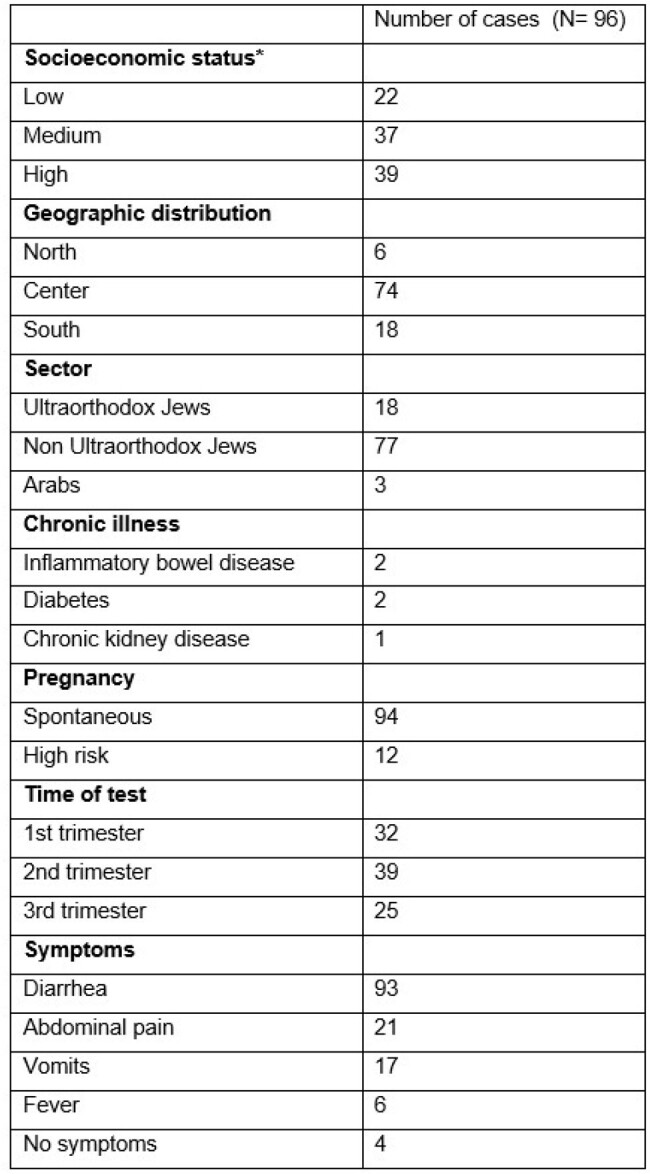
Baseline patient characteristics and symptoms, pregnant women infected with cryptosporidium

**Results:**

Between January 2020 and December 2023, 2512 pregnant women underwent PCR stool testing. Of them, 96 individuals were positive for Cryptosporidium, mostly in 2022 (Figure 1). Their median age was 31 years and ranged 23-42 years with medium-high socioeconomic status (37 and 39 women, respectively) (Table 1). Among those who tested positive, 93 out of 96 (96.7%) reported symptoms of diarrhea, and the infection was self-limiting in all cases, with none requiring specific treatment. In comparison to matched controls, no statistically significant differences were observed in the rates of intrauterine growth retardation, birth weight, delivery week, miscarriage, Apgar score, and head circumferences among the infants born to mothers with Cryptosporidium infection.

**Conclusion:**

This study sheds light on the clinical course of Cryptosporidium infection in pregnant women, emphasizing a generally favorable outcome without the necessity for therapeutic intervention. Further research may be warranted to explore the potential long-term effects and to refine preventive measures for this specific population.

**Disclosures:**

Shirley Shapiro Ben David, MD, GSK: Lecturer|Pfizer: Grant/Research Support|Pfizer: Lecturer

